# Oxidative responses and defense mechanism of hyperpigmented *P. aeruginosa* as characterized by proteomics and metabolomics

**DOI:** 10.17179/excli2018-1238

**Published:** 2018-06-13

**Authors:** Chadinee Thippakorn, Chartchalerm Isarankura-Na-Ayudhya, Supitcha Pannengpetch, Patcharee Isarankura-Na-Ayudhya, Nalini Schaduangrat, Chanin Nantasenamat, Virapong Prachayasittikul

**Affiliations:** 1 Center for Research and Innovation, Faculty of Medical Technology, Mahidol University, Bangkok 10700, Thailand; 2 Department of Clinical Microbiology and Applied Technology, Faculty of Medical Technology, Mahidol University, Bangkok 10700, Thailand; 3 Department of Medical Technology, Faculty of Allied Health Science, Thammasat University, Pathumthani 12120, Thailand; 4 Center of Data Mining and Biomedical Informatics, Faculty of Medical Technology, Mahidol University, Bangkok 10700, Thailand

**Keywords:** hyperpigmented P. aeruginosa, proteomics, metabolomics, stress response, melanin-like activity

## Abstract

*Pseudomonas aeruginosa* is known to produce multiple types of pigment which are involved in its pathogenicity and survival in certain environments. Herein, we reported the identification of *P. aeruginosa* dark-brown hyperpigmented (HP) strains which have been isolated from clinical samples. In order to study the role of these dark-brown containing secretions, alterations of metabolic processes and cellular responses under microenvironment of this bacterial pathogen, two-dimensional gel electrophoresis (2-DE) in conjunction with peptide mass fingerprinting (PMF) were performed. Protein spots showing the most significant differences and high spot optical density values were selected for further characterization. Fold difference of protein expression levels among those spots were calculated. Three major groups of proteins including anti-oxidant enzyme such as catalase, alkyl hydroperoxide reductase and also iron-superoxide dismutase (Fe-SOD), transmembrane proteins as well as proteins involved in energy metabolism such as ATP synthase and pyruvate/2-oxoglutarate dehydrogenase were significantly decreased in *P. aeruginosa* HP. Whereas, malate syntase and isocitrate lyase, the key enzyme in glyoxylate cycle as well as alcohol dehydrogenase were significantly increased in *P. aeruginosa* HP, as compared to the reference strain ATCC 27853. Moreover, the HP exerted SOD-like activity with its IC_50_ equal to 0.26 mg/ml as measured by NBT assay. Corresponding to secretomic metabolome identification, elevated amounts of anti-oxidant compounds are detected in *P. aeruginosa* HP than those observed in ATCC 27853. Our findings indicated successful use of proteomics and metabolomics for understanding cell responses and defense mechanisms of *P. aeruginosa* dark-brown hyperpigmented strains upon surviving in its microenvironment.

## Introduction

*Pseudomonas aeruginosa, *the most common non-fermentative gram-negative rod has been reported as an important cause of bacterial pathogenic infection for immunocompromised individuals and patients in hospital settings as a result of neutropenia, burns or cystic fibrosis (Morrison and Wenzel, 1984[30]; Poindexter and Washington, 1974[36]). In addition, *P. aeruginosa* is known to produce multiple types of pigment which are involved in its pathogenicity and survival in certain environments. Many *P. aeruginosa* strains elaborate the blue-green phenazine (Rada and Leto, 2013[39]) derived pigment as a major secretion. Pyocyanin and its derivatives exhibit antibiotic activity towards competitors by initiating the production of superoxide radical and other ROS. Furthermore, this pigment has been reported for decades, as one of the virulence factors of this organism. Other pigments secreted by *P. aeruginosa* include pyoverdine (yellow-green and fluorescent); an iron-binding compound for iron acquisition (Meyer, 2000[28]), pyorubin (red-brown), and pyomelanin (black brown) (Hunter and Newman, 2010[15]; Kodaka et al., 2003[21]). Unlike pyocyanin, which exerts cytotoxic effects toward other cells, pyorubin and pyomelanin from *P. aeruginosa* was discovered to have antioxidant activities pertaining to its survival in the environment (Orlandi et al., 2015[33]; Rodriguez-Rojas et al., 2009[40]). We have recently isolated from clinical samples, a hyperpigment (HP) producing *P. aeruginosa* strain with dark-brown melanin like pigment which is diffused through agar media. The growth supernatant of HP exerted protective effects to *E. coli* growth under paraquat induced oxidative stress conditions. To understand the role of this melanin like pigment production, cell responses, and metabolic changes that exhibited protective effects against free radical in oxidative stress conditions, were used in a proteomic approach in conjunction with peptide mass fingerprinting in this study. Moreover, characterizations of active molecules in the secre-tome of HP were performed using mass spectrometry analysis. The interrelation between differentially expressed proteins as well as the feasibility to understand cell expressions and responsibility for survival in certain environments have been discussed. Further understanding of the biological properties of microbial pigments and their derivatives will not only enrich our instinctual curiosity about colors, but also provide a scientific basis for therapeutic disarming of the pathogens or for borrowing these multifunctional molecules in pharmacologic applications. 

## Material and Methods

### Bacterial strains

*P. aeruginosa* ATCC 28753, PAO1 and dark-brown melanin-like pigment producing strain (isolated from clinical sources in Nakhon Pathom Hospital, Thailand) evaluated in this study were recovered in the Clinical Microbiology Laboratories of Faculty of Medical Technology, Mahidol University, Thailand.

### Growth media

Luria-Bertani (LB) broth, agar plates, and several defined media including glucose, cetrimide, urea, King P and King F medium were used for preliminary studies of growth and pigment production of *P. aeruginosa* ATCC 28753 and HP strains.

### Pigment characterization

Bacterial cultures of *P. aeruginosa* HP were grown in LB broth (10 g/L tryptone, 5 g/ L NaCl and 5 g/L yeast extract, pH 7.2) for 48 h at 37 °C. Dark-brown characteristics of the culture media were observed. Simultaneously, cells were separated from media by centrifugation at 8,000 rpm for 15 min and the supernatants were filtered through a 0.2 µm membrane filter. The absorption spectrum of the filtered dark-brown supernatant was measured using an ultraviolet-visible (UV-Vis) spectrophotometer at 25 °C.

### Growth characteristics on oxidative stress conditions

Cells were grown in 5 ml LB broth at 37 °C, in a shaking incubator at 130 rpm, overnight. 1:100 of overnight cultures were transferred into 50 ml broth and grown until OD reached 0.5 after which, 0.8 mM of paraquat was added in order to investigate the responsibility of cells in oxidative stress conditions. Growth characteristics of cells were observed and determined by monitoring the absorbance at 600 nm. 

### SOD like activity testing

Cultured media of *P. aeruginosa* ATCC 27853 and HP strains were collected. The supernatant containing pigments were separated by centrifugation. Subsequently, proteins were removed by TCA precipitation. Then, chloroform was applied for rapidly extraction of water soluble and un-soluble pigments. The dark-brown substances were solubilized in water and evaporated and the SOD-like activity was determined by NBT assay (Anandjiwala et al., 2008[2]). The reaction mixture consisted of 50 mM phosphate buffer (pH 7.6), 20 µg riboflavin, 12 mM EDTA, and NBT 0.1 mg/3 ml. Reaction mixture was activated by illuminating with different concentrations of the extracted bacterial growth supernatants for 150 s. Immediately after illumination, the absorbance of reaction mixture was evaluated at 590 nm and IC_50_ was then calculated. Methanol was used for blank reading while ascorbic acid was used as positive control.

### Protein preparation for proteomics analysis

500 µl of overnight cultures of *P. aeruginosa* HP and ATCC 27853 in LB broth were sub-cultured in 50 ml LB broth and grown for 18 h at 37 °C. Cells were collected by centrifugation at 8,000 rpm for 15 min at 4 °C and washed 3 times using 40 mM Tris, pH 8.0. Compared to cell pellets, 2 volumes of lysis buffer (8 M urea, 4 % CHAPS) freshly supplemented with 10 µl/ml protease inhibitor cocktail were added for inhibiting of endogenous protease. Cells were completely lysed by ultra-sonication on ice. Whole cell lysates were collected by centrifugation at 14,000 rpm for 30 min. The endogenous protein concentrations were quantified by Bradford protein assay using bovine serum albumin as a standard. The protein solution was stored at -20 °C for further analysis.

### Two-dimensional electrophoresis (2-DE)

13 cm IPG strips were rehydrated with 250 µl of rehydration buffer for 16 h at room temperature (8 M urea, 4 % CHAPS, 0.002 % bromphenol blue). Using cup loading technique, 300 µg of protein samples in 100 µl rehydration buffer containing 0.028 mg DTT, 1 % 3-10 IPG buffer, and 12 µl destreak were loaded into strips. Isoelectric focusing (IEF) was performed at 20 °C in Multiphor II as follows: 300 V, 1 mA, 5 W for 2 min; 3500 V, 1 mA, 5 W for 1.30 h; 3500 V, 1 mA, 5 W for 4.00 h. After IEF, the strips were stored at -80 °C until processing. Before starting the second-dimension, strips were equilibrated in SDS equilibration buffer (50 mM Tris-HCl, pH 8.8, 6 M urea, 30 % glycerol, 2 % SDS, brompenol blue) containing 1 % DTT for 10 min with shaking at 50 at room temperature and re-equilibrated in a similar buffer containing 2.5 % iodoacetamide instead of DTT for 10 min. Strips were continually placed on 12.5 % precast gels and then gel electrophoresis were carried out. Briefly, all gels were electrophoresed at 10 mA per strip for 30 min and then 20 mA per strip for 4.00 h. Gels were stained overnight by using colloidal Coomassie staining.

### Mass spectrometry and bacterial endogenous proteome identification

Mass spectrometry and peptide mass fingerprinting (PMF) analysis were performed as described (Isarankura-Na-Ayudhya et al., 2010[16]). Initially, protein spots were manually cut off the gels, transferred to microtiter plates, and then destained overnight in destaining solution (50 % methanol and 5 % acetic acid). In gel trypsin digestion was carried out using sequencing grade of modified trypsin (Promega, UK) and then extracted proteins from each spots were subjected to MALDI-TOF mass spectrometry for protein identification. MALDI-TOF mass spectrometer (Model ReflexIV, Bruker Daltonics, Germany) has been used based on peptide mass fingerprint mapping. Briefly, the extracted peptides were mixed with a solution of 10 mg/ml α-cyano-4-hydroxycinnamic acid (LaserBio Labs, France) in 66 % acetonitrile and 0.1 % trifluoroacetic acid (TFA) and spotted onto a 96-well target plate. The mass spectra were acquired in the positive ion reflector delayed extraction mode using approximately 200 laser shots. Peak lists were generated and transferred for further analysis using the XMASS software (Bruker Daltonics). Proteins identification were initiated by integrating of BioTool 2.0 software (Bruker Daltonics) and MASCOT 2.2 search engine (http://www.matrixscience.com/) to compare and calculate peptide identity score between the trypsin digested peptide fragment data with reference database, NCBInr. Search result scores greater than 59 was considered to be of significant difference (*p*-value < 0.05). 

### Database search for protein identification

To identify proteins, all mass spectra recorded on tryptic peptide derived from each spot were searched against SwissProt databases using MASCOT search program (www.matrixscience.com).

### Mass spectrometry and bacterial secretomic metabolome identification

The metabolites from filtered protein-free growth supernatants of *P. aeruginosa* were subjected to ESI-QTOF mass spectrometry for identification of active ingredients which exerts certain activity. The separation was carried out by DIONEX Ultimate 3000 HPLC (Dionex Softron, GmbH, Germany) and Acclaim PolarAdvantageII C18 (2.1 × 100 mm, 3 μm) column with Acclaim PolarAdvantageII C18 (3 × 10 mm, 5 μm) guard column. The injection volume was 5 μl. The column and autosampler temperature were maintained at 40 °C and 10°C, respectively. The detection was made by Bruker compact QTOF mass spectrometer (Bruker, Billerica MA, USA). MS signals in the m/z range of 50-1000 were separately acquired under positive-ion and negative-ion mode. The nebulizing gas pressure, the drying gas flow and the drying gas temperature was set at 2 bars, 8L/min, and 220 °C, respectively. The separation was performed at a flow rate of 0.4 mL/ min under a gradient program in which mobile phase A was composed of 0.1 % formic acid in water and mobile phase B was composed of 0.1 % formic acid in acetonitrile. The gradient program was applied as follows: t = 0 min, 1 % B; to 99 % B; t = 20 min.

### Database search for metabolomics identification

In order to identify the active metabolites in *P. aeruginosa* secretions, all mass spectral data were determined using Profiles Analysis Software. The identification of metabolites was carried out using the ChemSpider database (http://www.chemspider.com/).

## Results

### The production of dark-brown extracellular excretion of clinically isolated strain of P. aeruginosa

**Figure 1:**
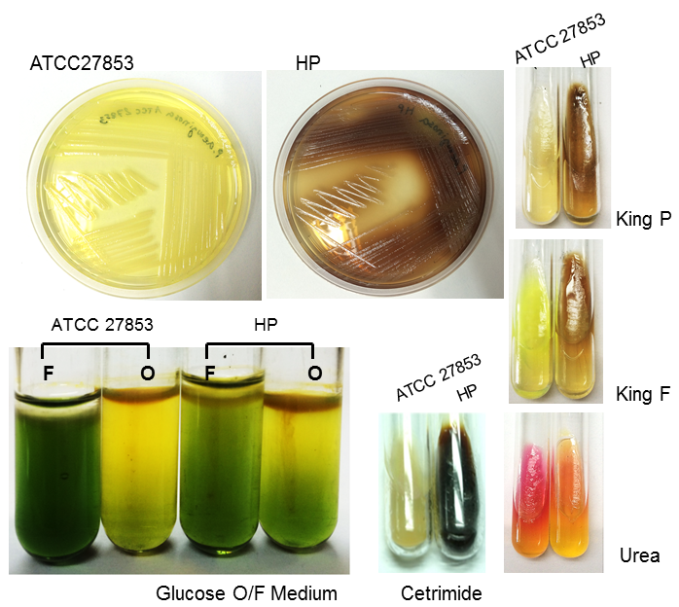
The comparison of pigment production in LB medium without any supplement from 24 h aerobic cultures of *P. aeruginosa* ATCC 27853 and *P. aeruginosa* high pigment clinically isolated strain HP. Brown zone was only observed around the colony of *P. aeruginosa *HP. Biochemical tests indicated varieties of pigment production based on the substrates utilized. Dark-brown pigment was observed on King P, King F, cetrimide and urea cultured medium but was not observed on glucose containing medium.

The clinical strain of *P. aeruginosa* was isolated from patients. When grown aerobically in LB medium, this clinically isolated *P. aeruginosa* (HP) highly produced extracellular, dark-brown, water-soluble substances into culture medium, leading to the change of media colour from pale yellow to dark-brown or black in both agar and liquid medium (Figure 1(Fig. 1)). In contrast, *P. aeruginosa* reference strain ATCC 27853 produced a green water-soluble pigment commonly known as pyocyanin. This phenomenon was time-dependent and occurred during mid to late exponential phase of growth in LB medium without supplementation. The pale-brown pigment was observed early from *P. aeruginosa* HP culture after 11 h of incubation. Optical intensity levels of this substance were increased over time. In addition, production of dark-brown pigment from *P. aeruginosa* HP was present in a bacterial culture on cetrimide and urea containing medium whereas it was not observed on glucose. This result implies that a biological synthesis of this pigment correlates with substrate utilization as it uses ammonia or nitrogen as the sole carbon energy source. Moreover, this dark-brown pigment was markedly produced from *P. aeruginosa* HP on growth of King P and King F media. This is in contrast to ATCC27853, which produced only yellow-green pigment on King F medium (Figure 1(Fig. 1)). 

The different excretions obtained from two varying strains of *P. aeruginosa* suggest a diverse mechanism inside cells as different substrate utilization were detected. Generally, cetrimide has been used to enhance pigment production. Elevated amount of dark-brown pigment released on cetrimide containing medium, together with growth characteristic on glucose and urea which are shown in Figure 1(Fig. 1), indicates a hyperpigment production of *P. aeruginosa* HP and its role in capability to survive in various conditions including nutrient limited conditions. 

### Increasing levels of cell survival capability in oxidative stress condition of dark-brown substance producing P. aeruginosa strain

To evaluate the capability of clinically isolated *P. aeruginosa* (HP) dark-brown pigment producer on oxidative stress, effect of superoxide radical induced by paraquat on growth characteristics of *P. aeruginosa* HP was investigated as compared to ATCC 27853 (the reference strain). After 3 h of incubation, 0.8 mM paraquat was added. A slight decrease in *P. aeruginosa* growth was observed for ATCC 27853 between 6-24 h. In contrast, growth of *P. aeruginosa* HP was seen to decrease when exposed to 0.8 mM paraquat until 12 h of incubation. A linear increase in growth of *P. aeruginosa *HP was observed after 12 h and correlated with increasing levels of the dark-brown pigment in medium, suggesting that production of dark-brown pigment helped the bacteria to increase its survival in oxidative stress conditions (data not shown).

Moreover, the induction of dark-brown pigment was rapidly detected when a cultured bacteria was exposed to paraquat as compared to unexposed bacteria, indicating that the production of dark-brown substance from *P. aeruginosa* is related to oxidative stress and might be involved in stress induced response mechanisms for protecting its growth in stressful conditions.

### Escherichia coli growth protection of a dark-brown containing supernatant from P. aeruginosa 

**Figure 2:**
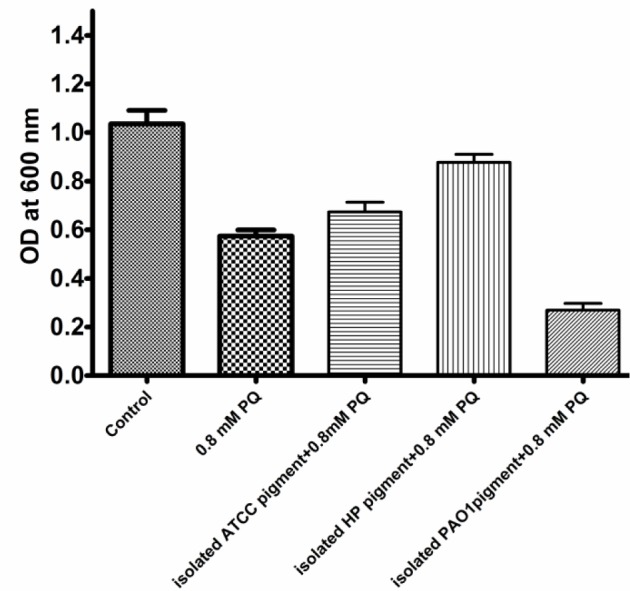
Protective effect of isolated dark-brown pigment for *E. coli* growth in paraquat (PQ) stress condition

To investigate the functional role of this substance, 24 h growth culture supernatant of three different strains of *P. aeruginosa* including *P. aeruginosa* ATCC 27853, PAO1, and HP were separated by centrifugation and filtered through 0.2 µm membrane filters. The filtered extracellular secretion containing supernatant was added into *E. coli* culture in the presence of 0.8 mM paraquat (PQ). Absorption at OD_600_ was used to determine the cell density of *E. coli* whereby a 50 % reduction of *E. coli* cell density was observed when cells were grown in paraquat induced-stress conditions. Filtered dark-brown supernatant was found to highly protect the growth of *E. coli* from superoxide radical (75 % protection). In contrast, a PAO1 filtered supernatant was found to enhance growth reduction of superoxide radical generated by paraquat as shown in Figure 2(Fig. 2). This finding suggested that the dark-brown substance containing supernatant produced by *P. aeruginosa *HP contains superoxide radical scavenging activity.

### Characterization of growth supernatant containing dark-brown substance produced from P. aeruginosa HP

The absorption measurement of dark-brown substance with radical scavenging activity was carried out using an ultraviolet-visible (UV-Vis) spectrophotometer. For the wavelength range from 300 to 700 nm, the peak absorption of this pigment occurs around 357 nm with a minor peak at 400 nm. 

**Figure 3:**
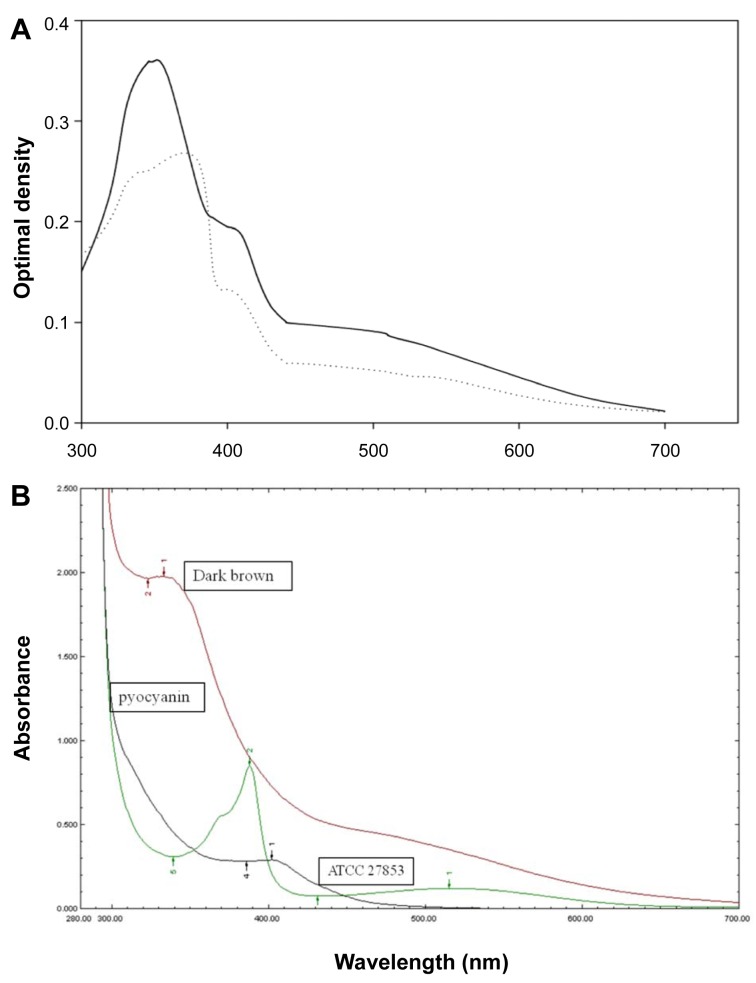
Absorption spectrum of dark-brown containing supernatant of *Pseudomonas aeruginosa* strain HP (straight line) and non dark-brown containing supernatant of ATCC 27853 (dot line). This measurement displayed a slightly increase in absorption from 700 nm to 430 nm and exponentially increases from 400 nm to 350 nm. The major absorption peak at 357 nm and minor peak (shoulder) at 400 nm were observed in *P. aeruginosa* HP supernatant, whereas the highest peak of *P. aeruginosa* ATCC 27853 supernatant was 380 nm with two shoulders at 320 and 400 nm (A). Different adsorption spectra was clearly detected when pigments were extracted and compared to pyocyanin (B).

The absorption is almost completely attenuated for wavelengths longer than 700 nm, the compound effectively absorbs solar UV wavelengths between 300 to 400 nm, whereas, the highest peak of *P. aeruginosa* ATCC 27853 supernatant was 380 nm with two shoulders at 320 and 400 nm. Absorbance characteristic of *P. aeruginosa* pigment is shown in Figure 3(Fig. 3).

### Identification of the endogenous protein expression profiling of P. aeruginosa HP compared with ATCC 27853

**Figure 4:**
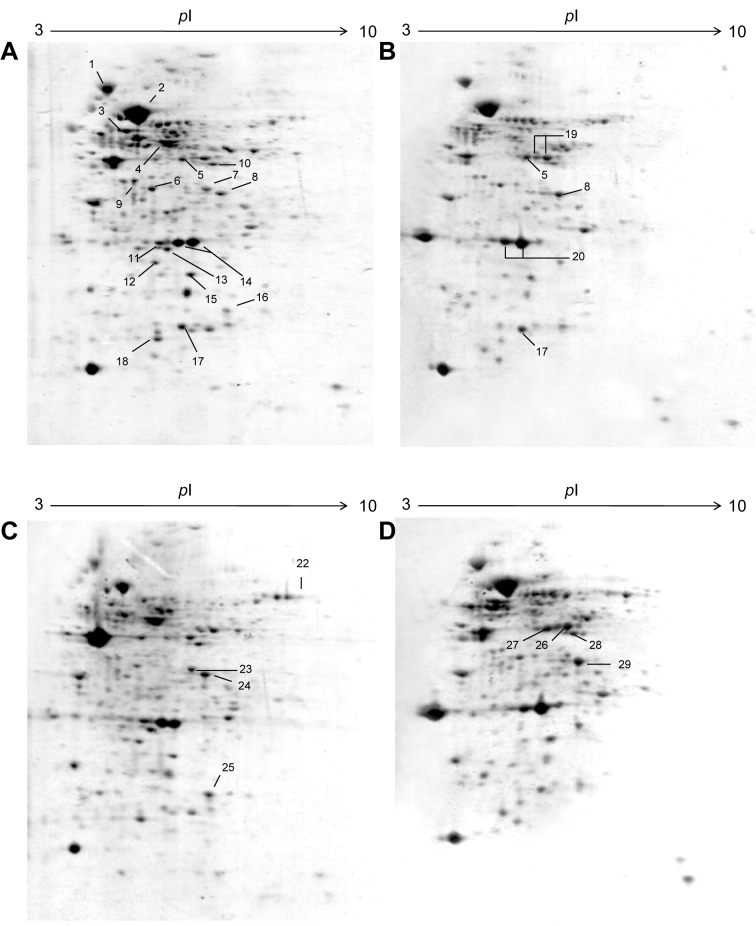
2D-PAGE image or the master maps representing protein profiles of *P. aeruginosa* ATCC 27853 (panels A and C), high pigment produced strain *P. aeruginosa* HP (panels B and D) in the absence of paraquat (panels A and B) and present of 0.8 mM paraquat (panels C and D) which was separated under pH ranges of 3-10. Numbers of protein spot denoted as identified protein are represented in Table 1.

**Figure 5:**
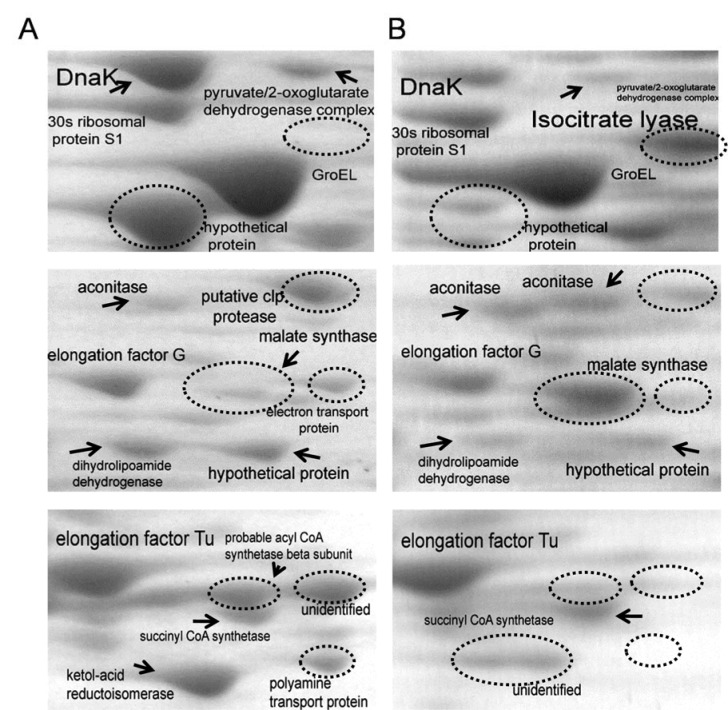
2D-Electrophoresis PAGE zoom into specific locations of ATCC27853 (A) and high pigment clinical isolated strain (B) in absence of paraquat revealing the difference in protein expression level of the reference strain as compared to high pigment.

Protein expression profiles of *P. aeruginosa* clinically isolated strain HP and reference ATCC 27853 were analyzed using high resolution two-dimensional gel electrophoresis (2-DE). Given the same amount of total protein sample tested, the optical density of most protein spots decreased in *P. aeruginosa* HP protein profile, as compared to ATCC 27853. Most protein spots fell in the pH 4-8 range, with only a small number of extremely acidic or basic proteins as shown in Figure 4(Fig. 4) and the zoom into specific location is presented in Figure 5(Fig. 5). Protein spots were further identified by MALDI-TOF mass spectrometry and bioinformatics database search engine MASCOT 2.2. The results are summarized in Table 1(Tab. 1). Twelve protein spots showing the most significant differences and high spot optical density values were selected. The fold differences of protein expression levels among those spots were calculated and summarized in Table 2(Tab. 2). In normal conditions, three major groups of proteins with ten protein spots including anti-oxidant enzymes such as catalase, alkyl hydroperoxide reductase, peroxidase and also iron-superoxide dismutase (Fe-SOD), transmembrane proteins as well as some proteins of energy metabolism including ATP synthase and pyruvate/2-oxoglutarate dehydrogenase were significantly under expressed, whereas two protein spots including malate synthase and isocitrate lyase were significantly highly expressed in *P. aeruginosa *HP, as compared to ATCC 27853.

### Metabolomics characterization of P. aeruginosa secretion reveals high abundance of antioxidant compounds in dark-brown hyperpigmented strains

Results from our findings reveal several bioactive molecules which exert different activities including anti-microbial activity, anti-oxidant, and anti-depressor as shown in Tables 3(Tab. 3) and 4(Tab. 4). Analyses of bioactive compounds are separated into positive and negative modes (Tables 3(Tab. 3) and 4(Tab. 4), respectively). Different types of compounds are observed between the different strains of *P. aeruginosa*. Compounds that exerted anti-microbial activities and derivatives were mostly detected in ATCC 27853 when compared to HP such as 3-Methyl-4-[(Z)-(4-methyl-5-oxido-1,2,5-oxadiazol-3-yl)-NNO-azoxy]-1, 2,5-oxadiazole 2-oxide with anti-schistosomal activity (Song et al., 2016[44]) is seen to be 5.66 folds greater in ATCC 27853 than HP. Surprisingly, more than 70 fold of differential secretion of 2-[4-(1,3-Benzodioxol-5-ylmethyl)-1-piperazinyl]-7-(4-methylphenyl)-7,8-dihydro-5(6H)-quinazolinone was observed. In contrast, lower amounts of anti-microbial activity exhibiting compounds were detected in HP. Instead, an elevated number of anti-oxidant molecules are observed. A 14 folds difference of Edaravone was observed to be expressed in the HP strain as compared to ATCC 27853. 

Not only Edaravone, but also 2-(1, 3-Dioxo-1H-benzo[de]isoquinolin-2(3H)-yl)-N-(2-methyl-1-propionyl-1,2,3,4-tetrahydro-4-quinolinyl)-N-phenylacetamide which is detected in high level in HP; up to 34 folds of differentiation was measured (Table 3(Tab. 3)). 

### Effect of free radical generated from paraquat on protein profiles of P. aeruginosa HP

Paraquat has been used universally for initiating the intracellular production of superoxide anion (O2˙^-^), which consequently leads to the inhibition of bacterial cell growth and induction of death. Our experimental study has been started by determining the differential expression of endogenous protein profiles of *P. aeruginosa *in the presence of paraquat. Results revealed that proteins from the reference strain of *P. aeruginosa *ATCC 27853 that were predominantly affected by oxidative stress generated by paraquat are the antioxidant enzymes and the proteins involved in energy production (Isarankura-Na-Ayudhya et al., 2010[16]). The slight increases of spot No. 12, 16 and the presence of spot No. 25 of iron-superoxide dismutase enzyme has been detected and shown in Figures 4A(Fig. 4) and 4C(Fig. 4). This indicates the first line of defense mechanism upon oxidative stress in normal situations. In contrast, the iron superoxide dismutase as well as peroxidase from protein profile of *P. aeruginosa* HP strain does not show any affect especially under oxidative stress induced conditions (Figures 4B(Fig. 4) and 4D(Fig. 4)) indicating the other first line defense mechanism as the probable role of the production of dark-brown pigment and its radical scavenging property found in *P. aeruginosa* HP. 

### SOD like activity testing on dark-brown extracted pigment

**Figure 6:**
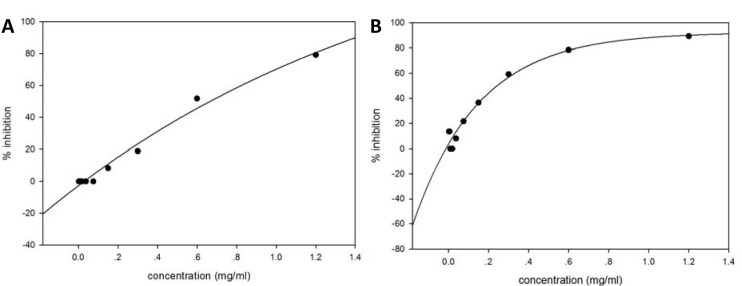
Free radical scavenging activity of extracted dark-brown pigment was determined using NBT assay. The dark-brown pigment extracted from *P. aeruginosa *shows the anti-oxidant activity effect which exerts its IC_50_ at 0.26 mg/ml (B) compared to pigment extracted from ATCC 27853 reference strain which exerts its IC_50_ at 0.67 mg/ml (A).

Assessment of superoxide radical scavenging activity has been performed based on the ability of dark-brown substance to inhibit blue formazan formation by scavenging the superoxide radicals generated in riboflavin-light-NBT system. Results are shown in Figure 6(Fig. 6). The dark-brown pigment extracted from *P. aeruginosa *shows the SOD like anti-oxidant activity effect which exerts its IC_50_ at 0.26 mg/ml as compared to the pigment extracted from ATCC 27853 reference strains which exert its IC_50_ at 0.67 mg/ml. This result correlates to the presence of several anti-oxidant molecules in secretion of *P. aeruginosa* HP strains and also the protective effect on the growth of *E. coli* under oxidative stress conditions.

## Discussion

Since a protective response to adverse environmental conditions, especially stress conditions were suggested as a functional role of the dark-brown or black pigment produced from microorganisms (Brakhage and Liebmann, 2005[5]; Kuznetsov et al., 1984[23]; Gomez and Nosanchuk, 2003[12]), the ability of microbes that can synthesize this dark-brown pigment or substance might be correlated with increased survival in environmental stresses. Melanin is one example of a dark-brown pigment produced by wide varieties of microorganisms (Bloomfield and Alexander, 1967[4]; Pavan et al., 2015[34]; Ruzafa et al., 1995[41]; Tang, 2009[47]). The properties of melanin pigment allow it to provide excellent protection from the deleterious effects of UV-A and UV-B light radiation. Melanin also protects microorganisms, such as bacteria and fungi, against stressors that involve cell damage by solar UV radiation or the generation of reactive oxygen species (Hamilton and Gomez, 2002[13]). Therefore, in many pathogenic microbes (for example, in *Cryptococcus neoformans*, a fungus that lives in the environment throughout the world) melanin appears to play an important role in virulence and pathogenicity by protecting the microbe against immune responses of the host (Alp, 2010[1]; Hamilton and Gomez, 2002[13]; Salas et al., 1996[42]; Williamson et al., 1998[49]). 

Pyomelanin is another type of melanin secreted from fungus and bacterial pathogens including *P. aeruginosa* (Hocquet et al., 2016[14]; Ketelboeter and Bardy, 2015[18]). This pigment has been reported to be derived from tyrosine catabolism. 

In this study we demonstrated the proteomics background of dark-brown hyperpigmented *P. aeruginosa* (HP) isolated from clinical samples which exhibit protective effects against free radical upon growth of *E. coli* under oxidative stress conditions. Corresponding with characterization of bioactive metabolites secreted from those pathogenic bacteria, all our findings reveal the possible role of dark-brown pigment in protective effects of cell growth and survival upon stress induced toxicity and cell death. In addition, our findings also demonstrated possible bioactive compounds that might be involved in stress response mechanisms. 

Besides the study of protein profile for the highly produced dark-brown pigment, clinically isolated strain of *P. aeruginosa* HP reveals the presence of key important enzymes, isocitrate lyase (ICL) and malate synthase (MS) of glyoxilase pathway (the unique enzymes specific for glyoxylate cycle) (Dunn et al., 2009[9]). The glyoxylate cycle is an anabolic metabolic pathway occurring in plants, bacteria, protists, fungi and several microorganisms, such as *E. coli, Pseudomonas spp.* and yeast. In microorganisms, the glyoxylate cycle allows cells to utilize simple carbon compounds such as acetate and compounds that promote acetyl CoA as a carbon source when complex sources such as glucose are not available (Figure 5(Fig. 5)). The glyoxylate cycle bypasses the steps in the citric acid cycle where carbon is lost in the form of CO_2_. The two initial steps of the glyoxylate cycle are identical to those in the citric acid cycle: acetate → citrate → isocitrate. In the next step, catalyzed by the first glyoxylate cycle enzyme, isocitrate lyase, isocitrate undergoes cleavage into succinate and glyoxylate. Glyoxylate condenses with acetyl-CoA which is catalyzed by malate synthase enzyme, yielding malate. Both malate and oxaloacetate can be converted into phosphoenolpyruvate, which is the substrate of phosphoenolpyruvate carboxykinase, the first enzyme in gluconeogenesis. The net result of the glyoxylate cycle is therefore, the production of glucose from fatty acids. 

Several reports indicate an important role for ICL and MS in many pathogens which are assumed to utilize fatty acid degradation products when growing in the host (Bloch and Segal, 1956[3]; Srivastava et al., 2008[45]) such as *Candida albicans* (Lorenz et al., 2004[25]), *Mycobacterium tuberculosis* (Murthy et al., 1973[31]), and some intracellular human bacterial pathogens such as *Salmonella enterica* serovar Typhimurium (Fang et al., 2005[10]; Kim et al., 2006[20]; Tchawa Yimga et al., 2006[48]), and *Brucella suis* (Kohler et al., 2002[22]). Many studies also observed that ICL and MS were reported to play a pivotal role during chronic infections in bacterial persistence (Fenhalls et al., 2002[11]; Dunn et al., 2009[9]). As present in this finding, most of the enzymes used to metabolize pyruvate, which is produced by glycolysis in the central metabolic pathway of *P. aeruginosa* ATCC 27853 remains unchanged. The main enzymes in the TCA cycle including isocitrate dehydrogenase, pyruvate/2-oxoglutarate dehydrogenase, and succinyl-CoA synthetase were still observed (data not shown). In contrast, the significant increase in expression levels of malate synthase (MS) and isocitrate lyase (ICL), key enzymes involved in the gyloxylate cycle, were detected (Figure 5(Fig. 5), Table 2(Tab. 2)). This indicated the adaptive metabolic changes of *P. aeruginosa* HP in energy metabolism for its survival inside the host which is probably a key role of *P. aeruginosa* chronic infection, such as a cystic fibrosis.

Among twelve potential protein spots showing a differentially expressed pattern, the most notable change was the decreased expression of antioxidant proteins including catalase, Fe-SOD, peroxidase and alkyl hydroperoxide reductase (AHP). Catalase is a common enzyme found in living organisms which are exposed to oxygen, where it functions to catalyze the decomposition of hydrogen peroxide to water and oxygen (Chelikani et al., 2004[6]). Catalase has one of the highest turnover numbers of all enzymes; one molecule of catalase can immediately convert millions of the toxic molecules of hydrogen peroxide to non-toxic molecules of water and oxygen per a second (Northrop, 1925[32]). The reaction of catalase in the decomposition of hydrogen peroxide is: 2H_2_O_2_ → 2 H_2_O + O_2_. On the other hand, Ahp is an NAD (P) H-dependent peroxidase, a member of the peroxiredoxins (prxs) family encoded by AhpC gene. Ahp was characterized as the bacterial organic peroxide detoxification system which catalyzes the reduction of organic peroxides to the corresponding organic alcohols and water molecules (Poole, 1996[37]). Ahp contains two cysteine residues and protects the cells against ROS. An internal redox-active disulfide bridge between the two cysteine residues are reduced by electron transfer from NADH via the FAD cofactor. The enzyme activity of Prxs was rapidly decreased by bursts of intracellular peroxide production (Wood et al., 2003[50]), in agreement with previous results of Chuang et al. (2005[8]) that the amount of AhpC in *H. pylori* greatly decreased after long-term oxidative stress. 

**Figure 7:**
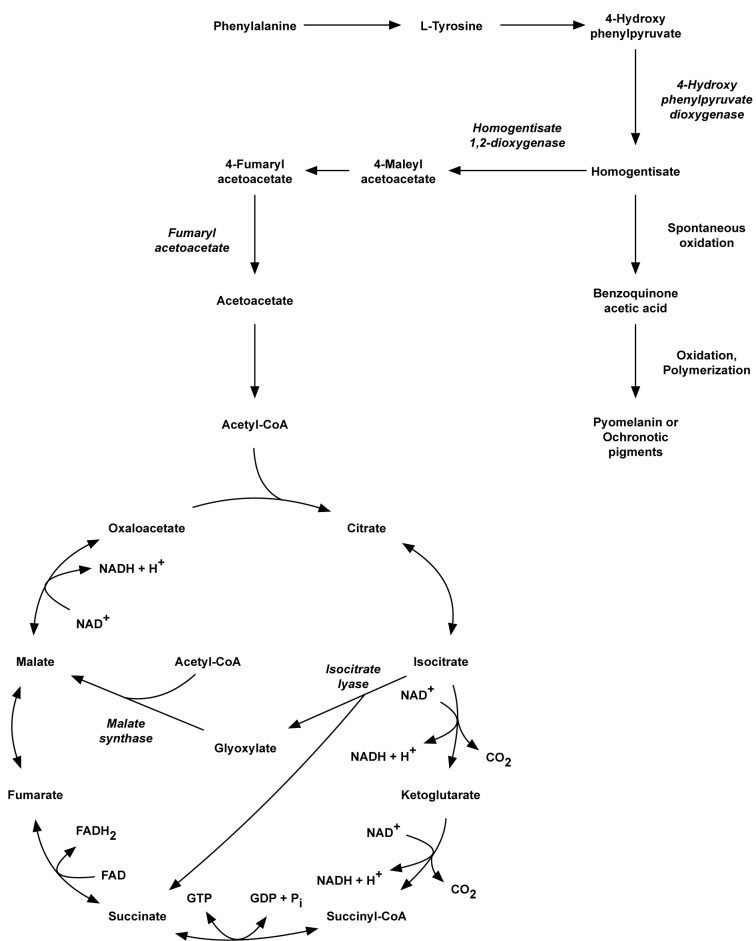
Enzymatic reactions of the glyoxylate and TCA cycles

Moreover, mutants that lack Ahp are indeed hypersensitive to growth inhibition by organic hydroperoxides, which are not substrates for catalase (Storz et al., 1989[46]). Thus, the loss of Ahp induced loss in activity of catalase (Seaver and Imlay, 2001[43]). Therefore, one reason for the decreased expression levels of catalase and alkly hydroperoxide reductase in protein profiles of clinically isolated strain *P. aeruginosa* HP as compared to ATCC 27853 is the long term exposure to oxidative stress generated from human defense mechanisms. Moreover, the finding of dark-brown pigments with protective activity against superoxide radical demonstrated one compensatory effect of this pigment in the radical scavenging mechanism of *P. aeruginosa*. Increase of extracellular anti-oxidant levels could be due to the compensatory effects of decreased intracellular anti-oxidant levels or could also be the consequent effect of glyoxylate bypass. The respiratory chain is known to be the primary source of H_2_O_2_ during aerobic growth on glucose, principally because of the auto-oxidization of NADH dehydrogenase initiated the generation of superoxide radical (Messner and Imlay, 1999[27]). Glyoxylate pathway is advantageous due to the reduction of using NADH-dehydrogenase enzyme initiated the attenuation of NADH+H+ and CO_2_ release finally lead to a decrease in H_2_O_2_ production , as shown in Figure 7(Fig. 7). The reduction in endogenous H_2_O_2_ generated by this mechanism bring on decreasing in protein expression level of alkyl hydroperoxide reductase, an NAD(P)H-dependent peroxidase enzyme and catalase in protein expression profile as revealed in our study.

Several bioactive compounds are identified in our work. Compounds that exerted antimicrobial activities along with derivatives were mostly detected in ATCC 27853 as compared to HP. The anti-schistosomal (Song et al., 2016[44]) compound, 3-Methyl-4-[(Z)-(4-methyl-5-oxido-1,2,5-oxadiazol-3-yl)-NNO-azoxy]-1,2,5-oxadiazole 2-oxide is produced up to 5.66 fold more in ATCC 27853 when compared to HP. Surprisingly, more than 70 fold of differential secretion of 2-[4-(1,3-Benzodioxol-5-ylmethyl)-1-piperazinyl]-7-(4-methylphenyl)-7,8-dihydro-5(6H)-quinazolinone were measured. Quinazolinone is a heterocyclic chemical compound possessing a wide spectrum of antimicrobial activities including anti-bacterial, anti-fungal, anti-viral activity etc. (Jafari et al., 2016[17]). 

In contrast, increasing number of anti-oxidant molecules produced from HP were also detected such as Edaravone. Edaravone is an intravenous medication used to help with recovery of strokes (Miyaji et al., 2015[29]). Even though the mechanism of action is unclear, it is known to contain antioxidant molecules (Kikuchi et al., 2013[19]; Lee and Xiang, 2018[24]; Petrov et al., 2017[35]). In addition, Edaravone was discovered to reduce paraquat activity and inhibit lipid peroxidation of alveolar epithelial cells under oxidative stress induced condition (Cheng et al., 2012[7]). Not only Edaravone but also 2-(1,3-Dioxo-1H-benzo[de]isoquinolin-2(3H)-yl)-N-(2-methyl-1-propionyl-1,2,3,4-tetrahydro-4-quinolinyl)-N-phenylacetamide was detected in high levels in HP with up to 34 fold of differentiation measured (Table 3(Tab. 3)). *N*-phenylacetamide is also known as acetanilide or acetanil (Malki et al., 2017[26]). Acetanilide is used as anti-oxidant molecules and is also used as a precursor in the synthesis of penicillin and other pharmaceuticals compounds (Prema and Sivakumar, 2015[38]).

## Conclusion

*P. aeruginosa,* a pathogenic bacteria, is considered to be a paradigm of antibiotic resistance development and a major cause of death in nosocomial infections. Herein, we have successfully demonstrated the use of proteomics and metabolomics technology in understanding the underlying mechanisms of stress responses in this pathogenic bacterium. Several bioactive compounds are identified with specific activities. Some compound including Edaravone is firstly discovering in secretome of microorganism as reported in our study. Therefore, further purification and characterization of the biological properties of those bioactive molecules and their derivatives will provide a scientific basis for therapeutic disarming of the pathogens or for implementing these multifunctional molecules in pharmacologic applications. 

## Notes

Chadinee Thippakorn and Chartchalerm Isarankura-Na-Ayudhya (Department of Clinical Microbiology and Applied Technology, Faculty of Medical Technology, Mahidol University, Bangkok 10700, Thailand; E-mail: chartchalerm.isa@mahidol.ac.th) contributed equally as corresponding authors.

## Acknowledgement

This work is supported by the Center of Excellence on Medical Biotechnology (CEMB), SandT Postgraduate Education and Research Development Office (PERDO), Office of Higher Education Commission (OHEC), Thailand.

## Figures and Tables

**Table 1:**
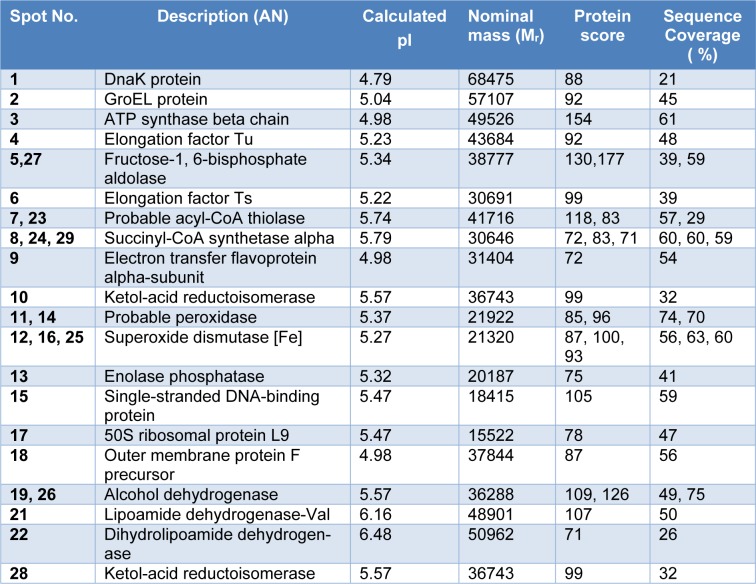
Proteins of *Pseudomonas aeruginosa* identified by mass spectrometry and peptide mass finger printing (PMF) analysis. Protein scores greater than 59 are considered to be significant (*p*-value < 0.05).

**Table 2:**
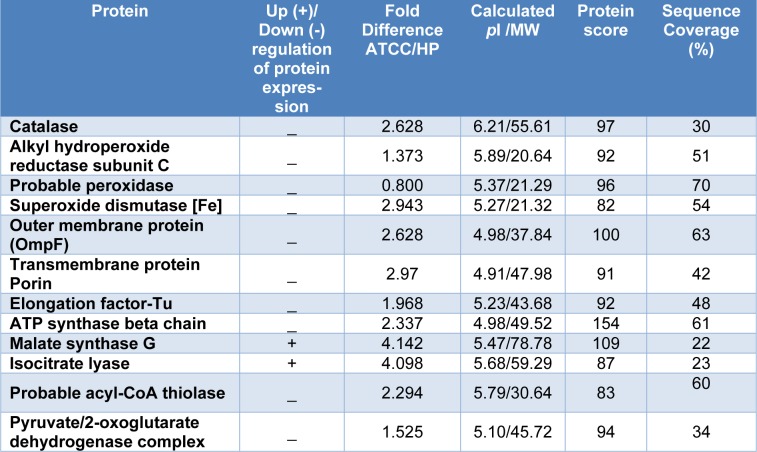
Changes of differentially expressed proteins of *P. aeruginosa* clinical isolated strain and reference strain following exposure to paraquat. Protein scores greater than 59 are considered to be significant (*p*-value < 0.05).

**Table 3:**
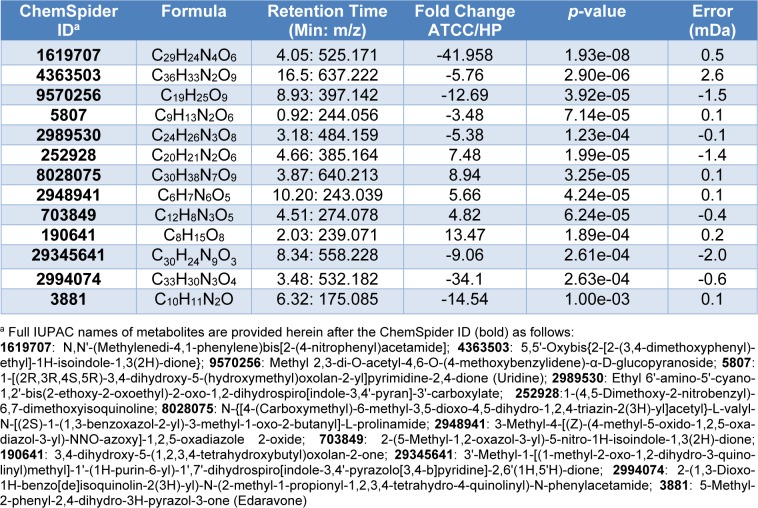
Identification of positive mode metabolites secreted from *Pseudomonas aeruginosa* identified by ESI/QTOF mass spectrometry. Expression levels are revealed as a comparison of fold change between ATCC and HP strains

**Table 4:**
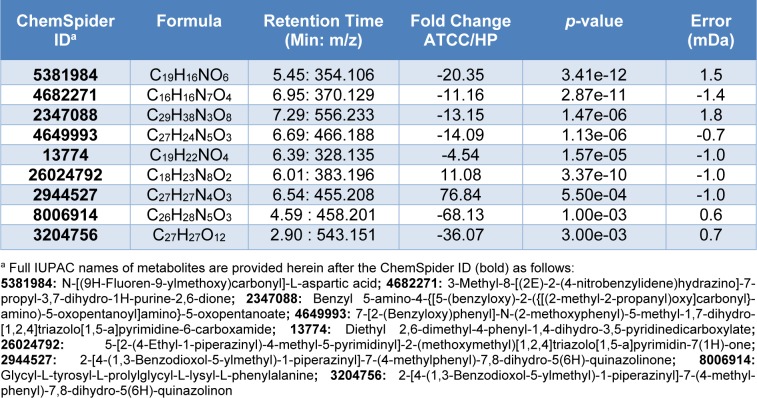
Identification of negative mode metabolites secreted from* Pseudomonas aeruginosa* identified by ESI/QTOF mass spectrometry. Expression levels are revealed as a comparison of fold change between ATCC and HP strains
